# Identification and Quantification of the Main Active Anticancer Alkaloids from the Root of *Glaucium flavum*

**DOI:** 10.3390/ijms141223533

**Published:** 2013-12-02

**Authors:** Lamine Bournine, Sihem Bensalem, Jean-Noël Wauters, Mokrane Iguer-Ouada, Fadila Maiza-Benabdesselam, Fatiha Bedjou, Vincent Castronovo, Akeila Bellahcène, Monique Tits, Michel Frédérich

**Affiliations:** 1 Plant Biotechnology and Ethnobotany Laboratory, Faculty of Natural Sciences and Life, University of Bejaia, Targa Ouzemour, 06000 Bejaia, Algeria; E-Mails: lbournine@student.ulg.ac.be (L.B.); bensalemsihem06@yahoo.fr (S.B.); fadilamaiza@yahoo.fr (F.M.-B.); fatihabedjou@yahoo.fr (F.B.); 2 Metastasis Research Laboratory, GIGA-Cancer, University of Liege, Institute of Pathology B23 avenue de l’Hôpital 3, B-4000 Liege, Belgium; E-Mails: vcastronovo@ulg.ac.be (V.C.); a.bellahcene@ulg.ac.be (A.B.); 3 Pharmacognosy Laboratory, CIRM, University of Liege, Institute of Pharmacy B36 avenue de l’Hôpital 1, B-4000 Liege, Belgium; E-Mails: jean-noel.wauters@ulg.ac.be (J.-N.W.); m.tits@ulg.ac.be (M.T.); 4 Marine Ecosystems and Aquacultural Laboratory, Faculty of Natural Sciences and Life, University of Bejaia, Targa Ouzemour, 06000 Bejaia, Algeria; E-Mail: imokrane@gmail.com (M.I.-O.)

**Keywords:** *Glaucium flavum*, Papaveraceae, HPLC-DAD, protopine, bocconoline, anti-cancer

## Abstract

*Glaucium flavum* is used in Algerian folk medicine to remove warts (benign tumors). Its local appellations are Cheqiq el-asfar and Qarn el-djedyane. We have recently reported the anti-tumoral activity of *Glaucium flavum* root alkaloid extract against human cancer cells, *in vitro* and *in vivo*. The principal identified alkaloid in the extract was protopine. This study aims to determine which component(s) of *Glaucium flavum* root extract might possess potent antitumor activity on human cancer cells. Quantitative estimation of *Glaucium flavum* alkaloids was realized by HPLC-DAD. *Glaucium flavum* effect on human normal and cancer cell viability was determined using WST-1 assay. Quantification of alkaloids in *Glaucium flavum* revealed that the dried root part contained 0.84% of protopine and 0.07% of bocconoline (*w/w*), while the dried aerial part contained only 0.08% of protopine, glaucine as the main alkaloid, and no bocconoline. *In vitro* evaluation of the growth inhibitory activity on breast cancer and normal cells demonstrated that purified protopine did not reproduce the full cytotoxic activity of the alkaloid root extract on cancer cell lines. On the other hand, bocconoline inhibited strongly the viability of cancer cells with an *IC*_50_ of 7.8 μM and only a low cytotoxic effect was observed against normal human cells. Our results showed for the first time that protopine is the major root alkaloid of *Glaucium flavum*. Finally, we are the first to demonstrate a specific anticancer effect of *Glaucium flavum* root extract against breast cancer cells, which can be attributed, at least in part, to bocconoline.

## 1. Introduction

*Glaucium flavum* (yellow hornpoppy) belongs to the family of Papaveraceae. *G. flavum* is an almost glabrous perennial herb with large yellow flowers and elongated capsule [1]. It spontaneously occurs along the entire Mediterranean coast (sandy land). It is also present in the Atlantic coast of Europe [2].

*G. flavum* is commonly referred as *Chelidonium* due to its morphological resemblance to *Chelidonium majus*. In France, this plant has synonyms: *Chelidonium fulvum Poir*. or *Chelidonium glaucium L*. [3]. *G. flavum* is used in different remedies depending on the regions. The powder seeds have been used as laxative [4]. The infusion of aerial part of *G. flavum* was used as antitussive [5]. The latex of the stem of this plant was used as antiseptic, cicatrizing agent and to heal minor sores and wounds [5]. In Algeria, this plant was used as detersive, hypotensive, and pectoral. The latex was employed locally to remove warts [6]. It has been demonstrated that the aerial part of *G. flavum* presented pharmacological activities including anti-inflammatory, analgesic and antipyretic [7], hypoglycemic [8], and antioxidant activity [9].

In a previous work, we studied the antitumor activity of *G. flavum in vitro* and *in vivo* [10]. We performed a preliminary cytotoxic screening *in vitro* towards human breast cancer cells of different extracts of *G. flavum*. The alkaloid root extract showed the most potent effect and using HPLC analysis, we demonstrated the presence of protopine as the major alkaloid in this extract. It has been reported earlier that the aerial part of this plant is very rich in isoquinoline alkaloids, principally the aporphine base *S*-(+)-glaucine (C_21_H_25_NO_4_) [11,12]. The amount of glaucine in yellow horn poppy grass has been previously determined by HPLC analysis [13]. There are however no reports comparing the analysis of the chemical composition and relative distribution of alkaloids in the root and aerial parts of the plant. In addition, to our knowledge, the analytical reports on roots are limited and poor data about the alkaloids composition of this plant part are available. Here, a comparative phytochemical study between the alkaloid extracts of aerial part and root of *G. flavum* was performed. In order to quantify the major active alkaloids beside the dry weight of the powder of aerial and root parts, HPLC quantification was monitored.

Hence, the present work therefore aims to identify and to quantify the compound(s) that are responsible of the antitumor effect of *G. flavum* root alkaloid extract, to perform a comparative phytochemical study between roots and aerial parts and to determine the occurrence of the main active alkaloids in these two parts of the plant.

## 2. Results and Discussion

**Figure 1.**
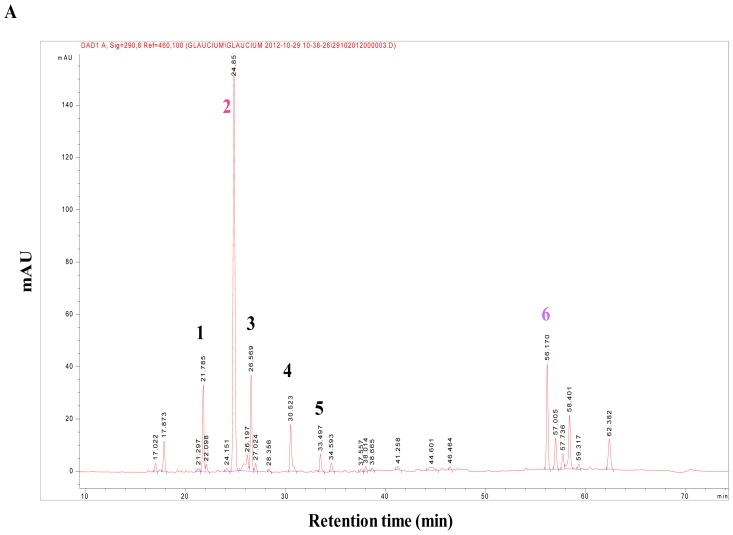

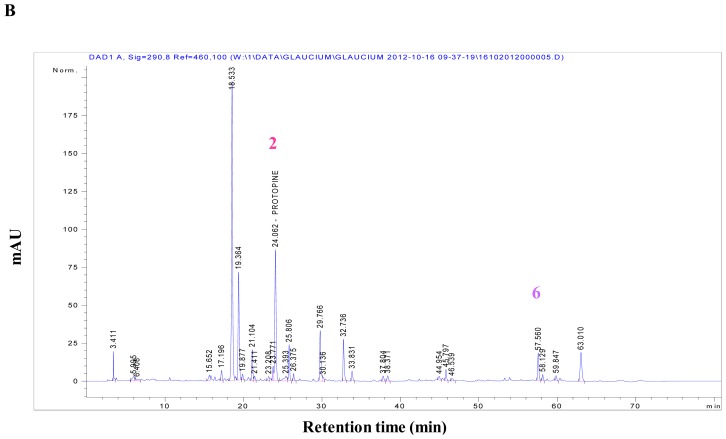
HPLC-DAD of alkaloidic extract (**A**) and methanolic extraction of the dry powder (**B**) of root of *G. flavum* at diode array UV-detection (λ = 290 nm). Peak identification: (**1**) magnoflorine, (**2**) protopine as major compound, (**3**) chelidonine, (**4**) sanguinarine, (**5**) chelerythrine, (**6**) bocconoline.

**Figure 2.**
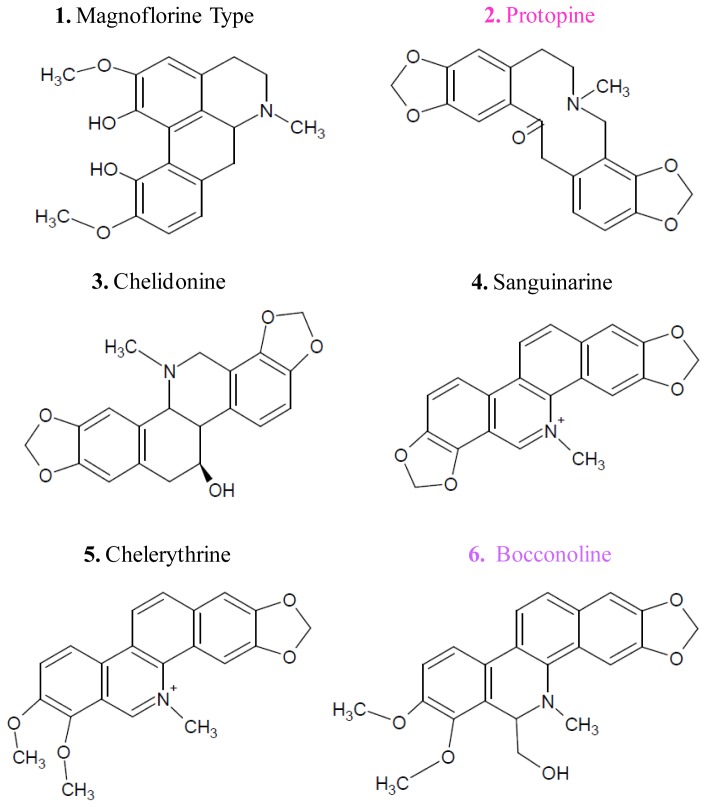
Molecular structure of identified alkaloids in the root alkaloid extract.

A preliminary phytochemical screening of *G. flavum* alkaloid root extract was realized by TLC and by comparison with reference standards. This analysis revealed the presence of alkaloids in the plant, using the Dragendorff reagent (data not shown). The HPLC-DAD analysis showed that alkaloids from the alkaloidic root extract could be divided in two main groups (Figure 1A). The first group was eluted with an approximative retention time of 35 min. It contained protopine as major compound (Peak **2**), magnoflorine (Peak **1**), chelidonine (Peak **3**), sanguinarine (Peak **4**), and chelerythrine (Peak **5**) (Figure 2). These compounds were identified by comparing their retention times, their UV and MS spectra with those of authentic standards injected under the same conditions.

**Figure 3.**
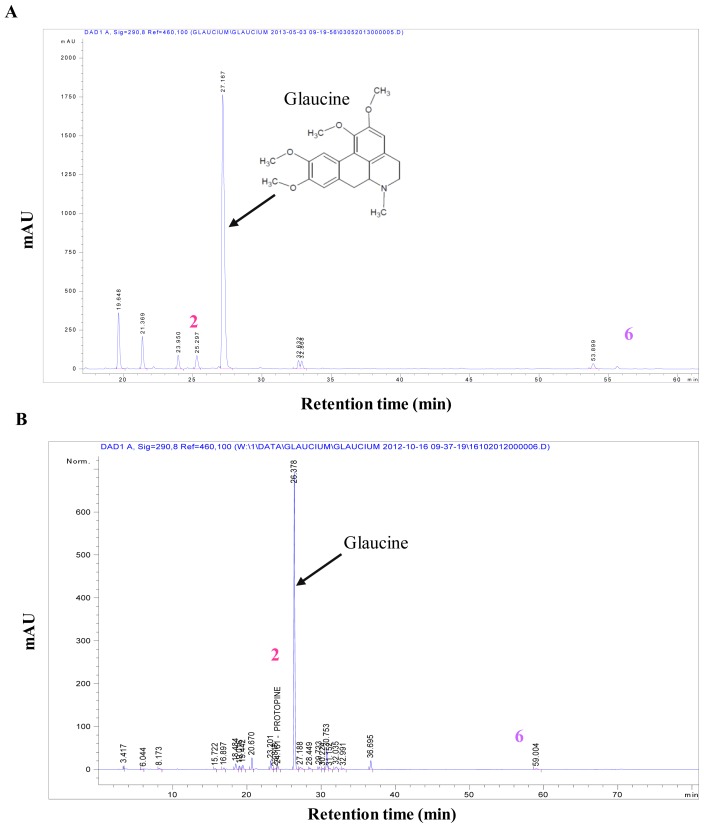
Representative HPLC-DAD chromatograms of alkaloidic extract (**A**) and methanolic extraction of the dry powder (**B**) of aerial part of *G. flavum*. Peaks (**2**) and (**6**) correspond to quantified compounds in plant (protopine and bocconoline, respectively). The major peak showed in section (**A**) and (**B**) correspond to glaucine.

Crystals of protopine were recovered by simple precipitation from the alkaloidic root extract in methanol. To identify this compound we used LC-MS in positive mode. The ESI-MS spectrum of this compound was found at *m*/*z* 354.13, which corresponded to the molecular mass of protopine. We confirmed this result by comparison of HPLC-DAD analysis of the obtained crystals and protopine standard (same retention time and UV spectra, data not shown). The qualitative and quantitative profiles of aporphinic alkaloids of *G. flavum* were completely different depending on the part of the plant. Quantitative determination of protopine in roots “alkaloid extract and dry powder” (Figure 1A,B) and in aerial part “alkaloid extract and dry powder” (Figure 3A,B) were carried out using HPLC-DAD.

The root part of *G. flavum* mainly contained protopine that reached a very high amount of 0.84% (in dried root) (Table 1). The concentration of the aporphinic alkaloids found in roots of Algerian *G. flavum* was higher than that reported previously in Brno, Czech Republic [14]. These differences and discrepancies could be explained by differences in method of analysis or geographical origin and the stage of development of the plant [15]. The aerial part of *G. flavum* had low levels of protopine 0.08% (in dried aerial part) while it contained a very high proportion of glaucine. The high concentration of glaucine in aerial part of *G. flavum* has been previously established [13]. Alkaloidic extracts of root and aerial part contained 41.60% and 3.86% of protopine (*w/w*), respectively (Table 1).

**Figure 4.**
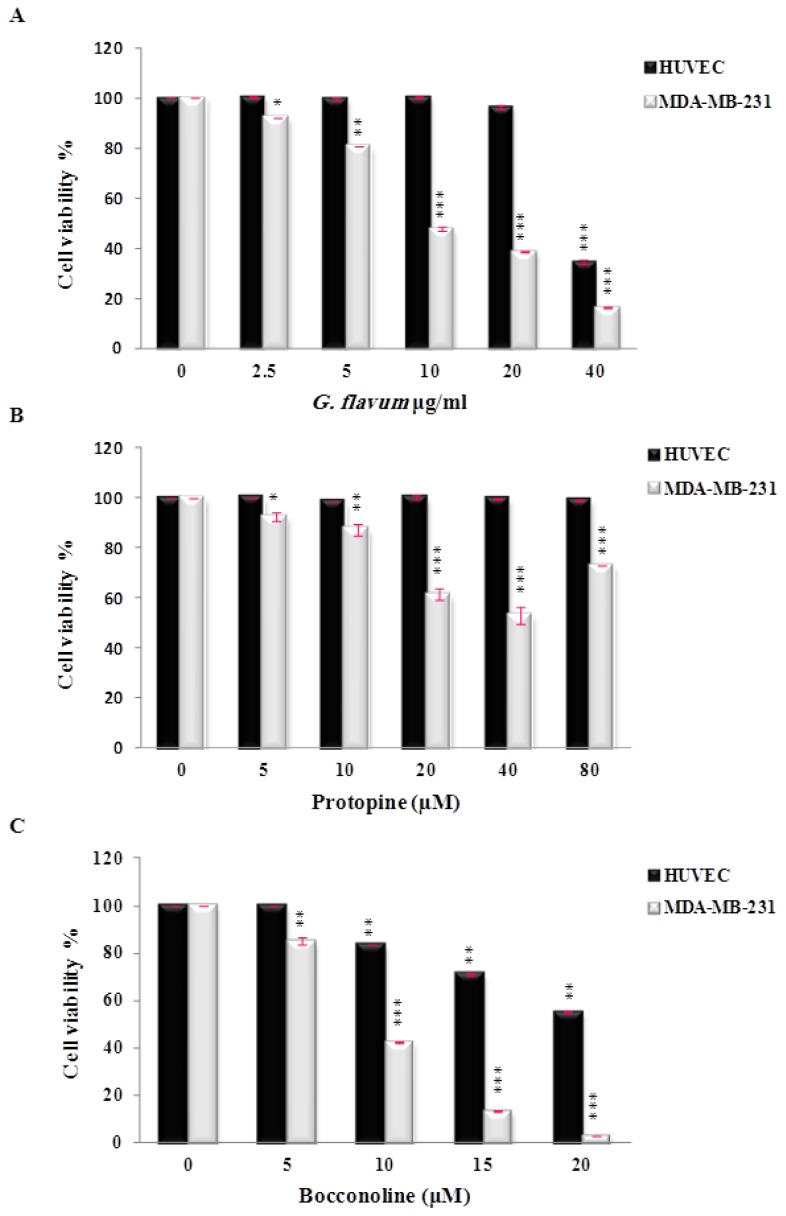
*In vitro* growth inhibitory activity of *G. flavum* alkaloids root extract (**A**), protopine (**B**), and bocconoline (**C**) against malignant human breast cancer cells (MDA-MB-231) and Human Umbilical Vein Endothelial Cells (HUVEC). Cells were treated with DMSO vehicle or the indicated concentrations of *G. flavum* alkaloids root extract, protopine and bocconoline for 24 h. Cell viability was determined using WST-1 assay and expressed as means ± S.E.M of three separate experiments (*n =* 3). * *p* < 0.05; ** *p* < 0.01; *** *p* < 0.001 compared with control group.

The effect of protopine on the viability of human breast cancer cells MDA-MB-231 and normal human cells (HUVEC) was evaluated *in vitro*. Protopine demonstrated a moderate cytotoxic effect against breast cancer cell lines and did not affect the growth of normal cells (Figure 4B). The inhibitory effect on cancer cell viability has been observed in the range of 20–40 μM. To our knowledge, only one study has reported the anti-proliferative effect of protopine using human prostate cancer cells [16]. These authors used concentrations of protopine ranging from 3 to 50 μM to evaluate the effect of protopine on cancer cell viability. They demonstrated that protopine caused tubulin polymerization, leading to Cdk1 activation and mitotic arrest of the cell cycle that finally triggered mitochondria-mediated apoptotic signaling pathwAays [16]. Interestingly, we noticed that protopine used over 40 μM appeared to promote cancer cell viability (Figure 4B). Based on the available literature, at least two mechanisms can be proposed to explain this result. A previous study reported that protopine exerted a cellular protective effect on hydrogen peroxide-induced apoptosis and oxidative stress in rat tumor cells [17]. In this study, the authors proposed that the protective effect of protopine could be attributed firstly, to an anti-apoptotic mechanism including: (a) a decrease of the intracellular Ca^2+^ content via directly blocking Ca^2+^ channels; (b) an increase of the mitochondrial membrane potential, and (c) an inhibition of caspase-3 expression; and secondly, through an antioxidant mechanism by enhancing the activity of the intracellular antioxidant enzymatic system. These authors indicated that this antioxidant ability may be attributed to pyrocatechol, a metabolite of protopine [17]. In light of these results, we propose that protopine at high concentration may promote the viability of human breast cancer cells through similar anti-apoptotic and/or an antioxidant mechanisms. Ongoing studies will help to determine the precise mechanism(s) of this potential dual role of protopine on cancer cell survival and growth. Furthermore, the determination of the precise cut off value and concentration limits of protopine will be necessary before its use as an anti-neoplastic agent.

These findings led us to search for another major compound, which could explain the global antitumoral effect of *G. flavum* alkaloid extract. Thus, we decided to isolate and identify the major compound of the second group of alkaloids of the root extract using preparative HPLC. This group of compounds was eluted at a retention time close to 60 min in the HPLC separation; it contained one major compound (Peak **6**) (Figure 1A). This compound was eluted at 56.61 min. Its molecular formula was determined to be C_22_H_21_NO_5_ by HR-ESIMS analysis ([M+Na]^+^ at *m*/*z* 380.15). The identification of compound (**6**) was determined by NMR analysis (^1^H, ^13^C, COSY, HSQC and HMBC). The 2D NMR has never been recorded previously and is presented here for the first time (Supplementary Figures 1–5). The compound (**6**) was identified as bocconoline. The structure of bocconoline is presented in Figure 2. Previously, the ^1^H and ^13^C NMR data for bocconoline were reported in *Rutaceae* (bark of *Zanthoxylum davyi* and *Fagara mayu*) [18,19]. Hence, the quantitative estimation of bocconoline was approached in alkaloid extracts and in dry powders of root and aerial parts of *G. flavum* using HPLC-DAD (Figure 3A,B and Figure 1A,B). We determined that the underground part of *G. flavum* contained bocconoline at a concentration of 0.07% (in dried root), and at a concentration of 4.43% (*w/w*) in alkaloid extract. This compound was completely absent from the dry powder and alkaloid extract of the aerial part (Table 1).

The potential cytotoxic effect of bocconoline on cancer cells has never been investigated. The viability test showed that it has a potent antiproliferative activity against MDA-MB-231 human breast cancer cells in a dose-dependent manner (Figure 4C), with an *IC*_50_ value of 7.8 μM. In this range of concentrations, bocconoline did not affect significantly the growth of normal human cells (HUVEC). This profile is similar to *G. flavum* alkaloid root extract, which exhibited a selective effect toward cancer cells without affecting normal cells. Altogether our data suggest that bocconoline is mainly responsible of the anti-proliferative effect of *G. flavum* alkaloid root extract (Figure 4A–C). More studies are needed to determine the molecular mechanism of action of bocconoline and to evaluate its anticancer activity *in vivo*.

## 3. Experimental Section

### 3.1. Standards and Chemicals

Protopine, chelerythrine, and chelidonine were obtained from Extrasynthese (Lyon, France) and sanguinarine was purchased from Sigma-Aldrich (St. Louis, MO, USA). All references had a purity ≥98.0% by HPLC-DAD. HPLC grade acetonitrile and methanol were purchased from Merck (Darmstadt, Germany). Trifluoroacetic acid (TFA) of HPLC analytical-grade was purchased from Sigma-Aldrich (St. Louis, MO, USA). The ultra pure water used for the HPLC analysis was obtained from a Millipore system (Milli-Q RG) (Millipore, Molsheim, France). All other solvents were of analytical grade and were purchased from Merck (Darmstadt, Germany).

### 3.2. Instrumentations

Chromatographic quantification was performed using an Agilent 1100 HPLC Series system (Agilent, Santa Clara, CA, USA) consisting of a degasser (G1379A), a high-pressure quaternary pump (G1311A), an autosampler (G1313A), a thermostatic compartment (G1316A), and a diode array detector (G1315A). Preparative High Performance Liquid Chromatography (Varian System) is consisting of a high-pressure binary pump (PrepStar), a diode array detector (ProStar), and an automatic fraction collector (440-LC). To determine the structure of isolated compounds we used various spectroscopic techniques. NMR spectra were recorded in CDCl_3_ using a Bruker Avance II 500 MHz spectrometer (Messtechnik GmbH, Karlsruhe, Germany) equipped with a cryoprobe and using TMS as the internal reference. Mass spectrometry (MS) was performed with a micromass ESI-Q-TOF II instrument (supplied by Waters, Milford, MA, USA) using ESI-ionization in positive mode. The identity of the purified compounds was assessed by comparison of their NMR and MS data with data from the literature. The analysis of extract and isolated compounds were conducted using thin layer chromatography precoated Si gel F254 (Merck, 1.05735) plates.

### 3.3. Plant Material

The aerial parts and roots of *G. flavum* were collected in the littoral area in Tichy, province of Bejaia (Algeria), in June 2010, during the flowering period. These samples were authenticated by the botanists of University of Bejaia. A voucher specimen Nr 2011/0614 was left in the herbarium of the Laboratory of Pharmacognosy of University of Liege. The plant materials were cleaned and air-dried at room temperature. Both parts were ground to a fine powder and passed through a 63 mesh sieve, to provide homogeneous powder for the analysis. Powdered materials were maintained at room temperature, and protected from light until required for analyses.

### 3.4. Extraction

#### 3.4.1. Alkaloid Extract

The extraction of alkaloids from *G. flavum* was carried out as following: the powdered root (10 g) was extracted with methanol (100 mL) using soxhlet apparatus. Methanol was evaporated under reduced pressure and the residue was taken up in 2% hydrochloric acid (50 mL), and then filtered through GHP 0.45 μm filter (Waters Cooperation, Milford, MA, USA). The filtrate was adjusted to pH 8 with aqueous ammonia and extracted three times with dichloromethane (25 mL). The resulting extract was dried over MgSO_4_ and the solvent evaporated to obtain the crude alkaloidic extract (0.2 g) [20].

#### 3.4.2. Methanolic Extraction

The powdered aerial and root parts of *G. flavum* (1 g) were percolated with methanol (100 mL). The solution was filtered (membrane filter 0.45 μm), giving the sample before HPLC injection for the quantification of the main active alkaloids in the dry powder of the root and aerial part of *G. flavum*.

### 3.5. HPLC-DAD Analysis and Quantification of Protopine and Bocconoline in G. flavum Extracts

#### 3.5.1. HPLC Conditions

The separation of *G. flavum* alkaloids was carried out using a Polaris Amide C_18_ Column (250 × 4.6 mm i.d., 5 μm particle, Varian, Palo Alto, CA, USA), which was maintained at 25 °C. The mobile phase was composed of a gradient of solution A (trifluoroacetic acid 0.05% in water) and solution B (acetonitrile) programmed as follows: equilibration time 15 min at 100% A and linear gradient elution: 0 min 100% A; 1 min 97% A; 45 min 60% A; 55 min 40% A; 65 min 40% A and 66 min 100% A. The flow rate was 1 mL/min and the injection volume was 10 μL. The run time of each analysis was 80 min. Spectral data from all peaks were recorded in the range of 200–400 nm, and chromatograms were recorded at 290 nm. Protopine was isolated and identified as described previously [10]. The Agilent ChemStation (vA09.03) and DataAnalysis (v5.3) software were used for the acquisition and analysis of chromatographic data. Bocconoline was isolated from the alkaloids root extract of *G. flavum*. It was fractionated by PrepHPLC on a Luna PFP (pentafluorophenyl) column (250 × 4.6 mm, 5 μm, phenomenex^®^). A total of 376 mg of the dichloromethane extract were dissolved in methanol (7 mL) and filtered through an Acrodisc PSF GXF/GHP 0.45 μm filter and injected in PrepHPLC. The mobile phase consisted of TFA 0.05% (A) and methanol (B), which was applied in the following gradient elution: 0 min 80% A; 45 min 20% A and 80 min 0% A. Flow rate was 25 mL/min. The software used for the acquisition and analysis of chromatographic data in PrepHPLC was the Modular HPLC System Galaxie. The isolated compound (bocconoline) was identified by MS and ^1^H, ^13^C NMR, COSY ^1^H–^1^H, HSQC, HMBC.

#### 3.5.2. Preparation of Standard Solutions

Standard solutions were prepared in methanol for protopine (4–16 mg/100 mL) and for bocconoline (0.65–3.25 mg/100 mL). The solution was filtered through a 0.45 μm membrane filter disc before HPLC injection. The peak area of protopine and bocconoline were plotted against the concentration to obtain the calibration graph. A regression analysis was realized and the correlation coefficients were calculated (protopine *r*^2^ > 0.99996 and bocconoline *r*^2^ > 0.99983).

### 3.6. Cell Viability Inhibition

#### 3.6.1. Cell Lines

Human Umbilical Vein Endothelial Cells (HUVEC) were isolated and maintained in culture as described previously [21,22]. Human breast cancer cells MDA-MB-231 (HTB-26, ATCC) were grown in Dulbecco’s Modified Eagle’s Medium (DMEM), supplemented with 10% of fetal bovine serum and 1% l-glutamine. All the cells were cultured at 37 °C in a humidified atmosphere and 5% CO_2_.

#### 3.6.2. Cell Viability Analysis

Cell viability was determined using the cell proliferation reagent WST-1 assay according to the manufacturer’s instructions (Roche, Basel, Switzerland). All analyzed cells were seeded to obtain 50% of confluence after 24 h of incubation in 96-well plates and then treated with serial dilutions of isolated compounds: bocconoline and protopine (0–20 and 0–80 μM, respectively). Cells were then incubated with WST-1 reagent for 4 h. After this incubation period, the formazan dye formed was quantified with a scanning multi-well spectrophotometer at 450 nm. The measured absorbance directly correlates to the number of viable cells. Percentages of cell survival were calculated as follows:

(1) % cell survival=(absorbance of treated cells/absorbance of cells with vehicle solvent)×100

The half inhibitory concentration (*IC*_50_) was calculated from the dose-response curve obtained by plotting the percentage of cell survival *versus* the concentration of compounds used.

### 3.7. Statistical Analysis

Results are expressed as mean ± S.E.M. of at least three independent experiments. Student’s *t*-test was used to compare the difference between each treated group to the control. *p*-Values < 0.05 were considered as significant.

## 4. Conclusions

We demonstrate here for the first time that protopine is the major alkaloid in *G. flavum* root extract and we confirmed that glaucine is the major compound in the aerial part as described in the literature. High-performance liquid chromatography (HPLC-DAD) has been used to quantify active alkaloids in *G. flavum* aerial part and root extracts. We showed that the chromatogram of roots contained two main groups of alkaloids. The first group contained protopine as the major compound, with a concentration of 41.6% (*w/w*) in the alkaloid root extract. The cytotoxic effect of this alkaloid on viability of breast cancer was evaluated and we have observed no cytotoxic effect against human normal cells. The main alkaloid of the second group was identified as bocconoline (4.43% *w/w*). The cytotoxic effect of this alkaloid against human breast cancer MDA-MB-231 cells was dose dependent with an *IC*_50_ = 7.8 μM and only low cytotoxicity was observed toward normal cells. These findings led us to suggest that the specific anticancer effects of *G. flavum* extract could be attributed, at least in part, to bocconoline, which is the second major alkaloid of the root. Further investigations are needed to confirm the pharmacological activity of the major alkaloids present in *G. flavum* extract in animal models, to assess the safe use of the plant, which could lead to the potential development of an effective cancer chemotherapy agent.

## Supplementary Information



## Figures and Tables

**Table 1.** Content (%) of alkaloids **2** (protopine) and **6** (bocconoline) in *G. flavum* root and aerial parts.

Plant part	Root *	Aerial part *	Root alkaloid extract	Aerial part alkaloid extract
***Protopine***	0.84%	0.08%	41.60%	3.86%
***Bocconoline***	0.07%	-	4.43%	-

(*) Amount in the dried plant was calculated using a methanolic extraction; (-) Not detectable.
