# Supplementary material for: Identification and Quantification of the Main Active Anticancer Alkaloids from the Root of Glaucium flavum
Source: Int J Mol Sci. 2013 Dec 2;14(12):23533–44. doi: 10.3390/ijms141223533 (PMC3876061; doi:10.3390/ijms141223533)
Supplement: Supplementary file 1 [file ijms-14-23533-s001.pdf]

Supplementary Information

Figure S1. ^{1}H-NMR Spectroscopic data of compound **6** (500 MHz, CDCl3).

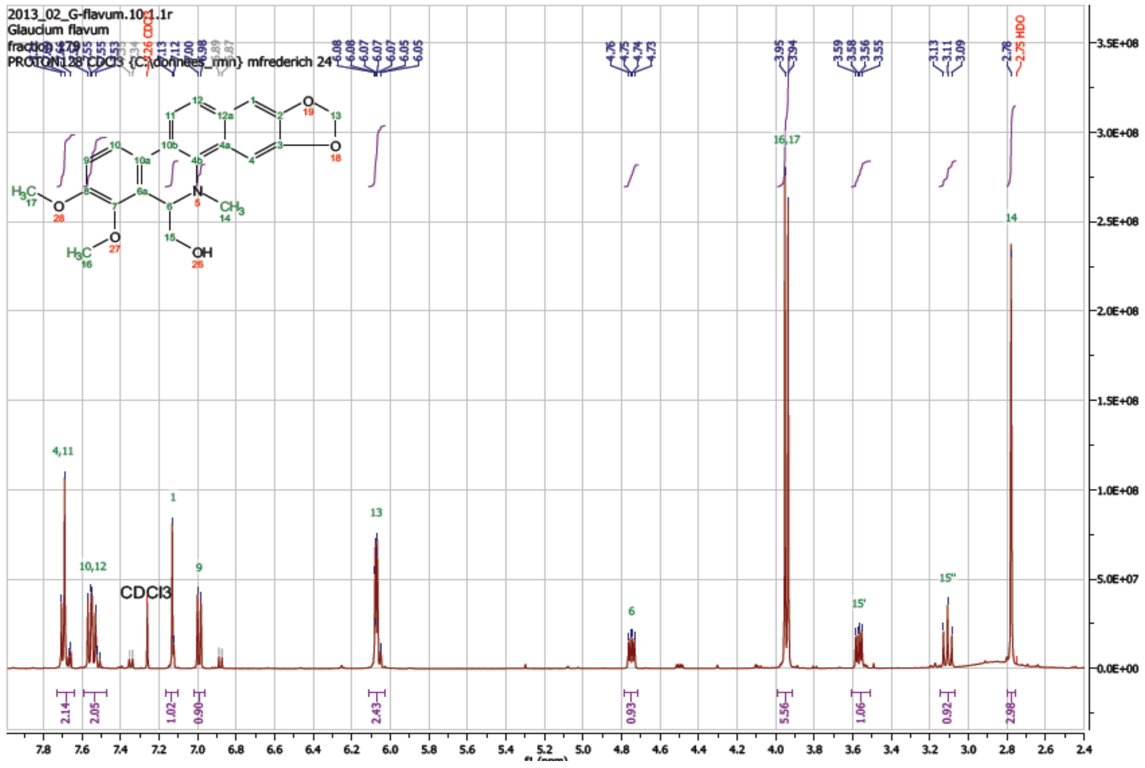

Figure S2. 13C-NMR Spectroscopic data of compound 6 (500 MHz, CDCl3).

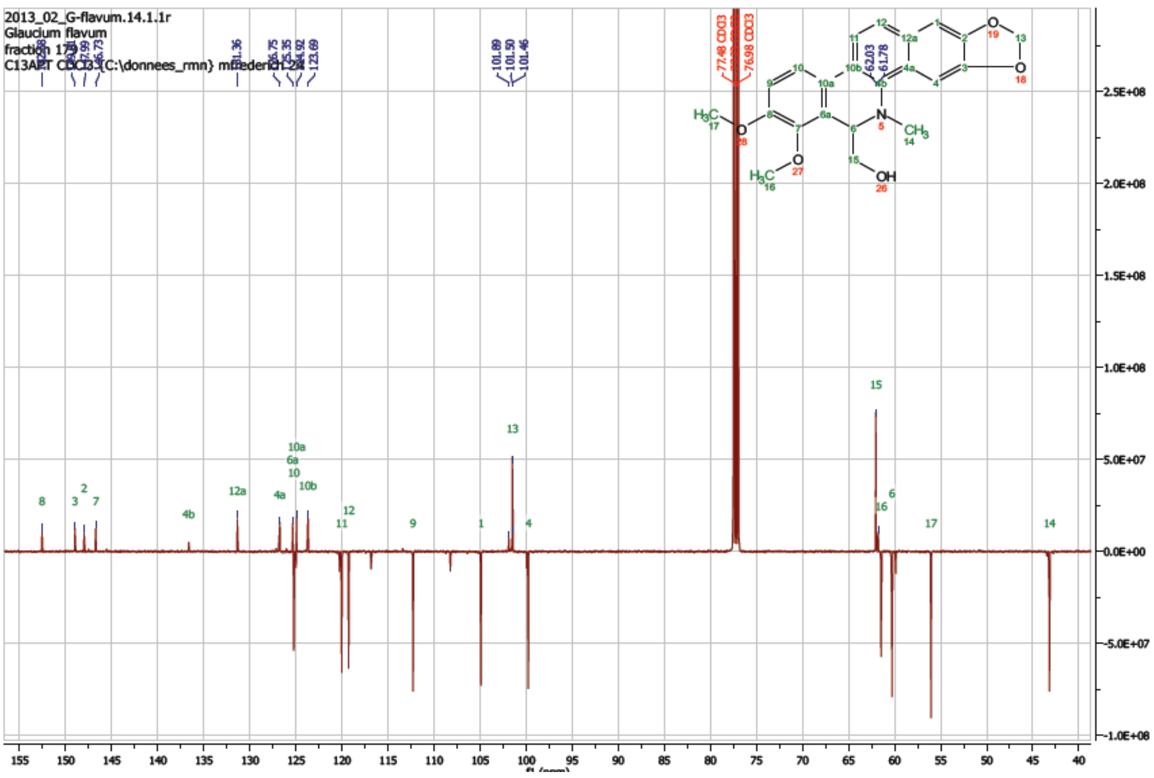

Figure S3. ^{1}H–^{1}H COSY-NMR Spectroscopic data of compound **6** (500 MHz, CDCl3).

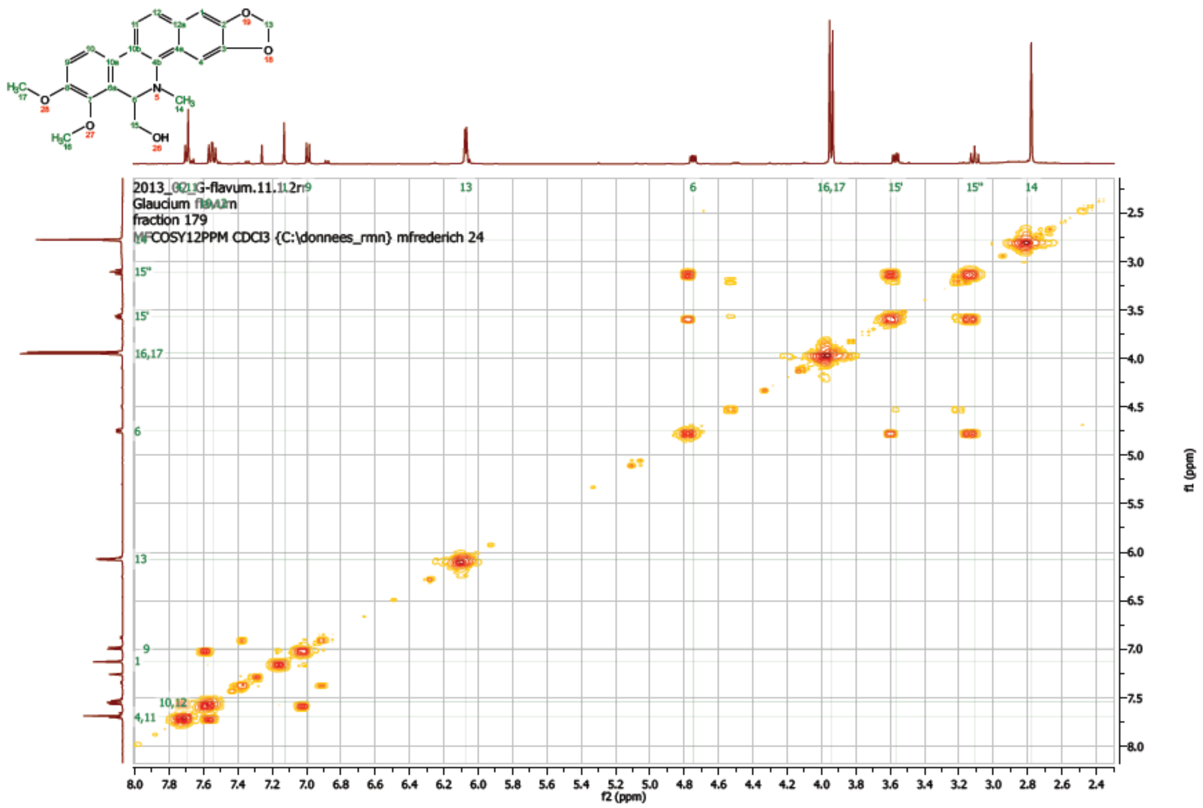

Figure S4. HMBC-NMR Spectroscopic data of compound **6** (500 MHz, CDCl3).

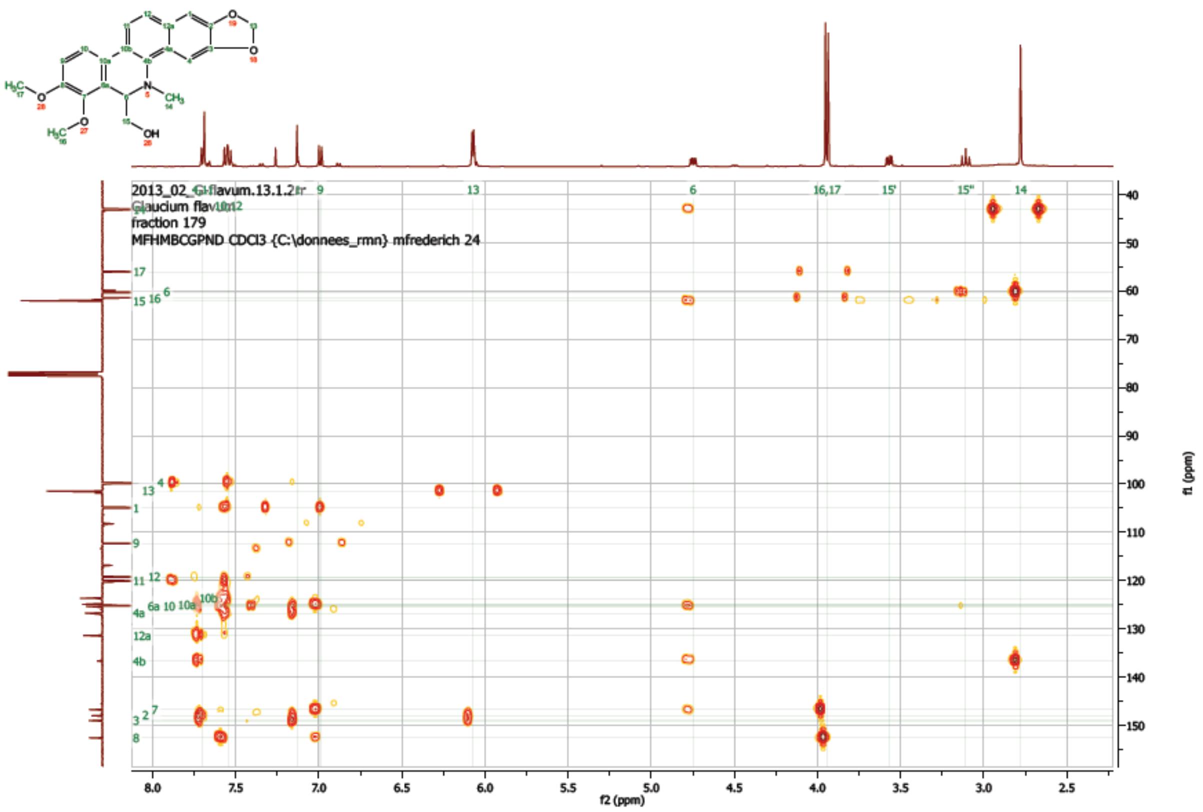

Figure S5. HSQC-NMR Spectroscopic data of compound **6** (500 MHz, CDCl3).

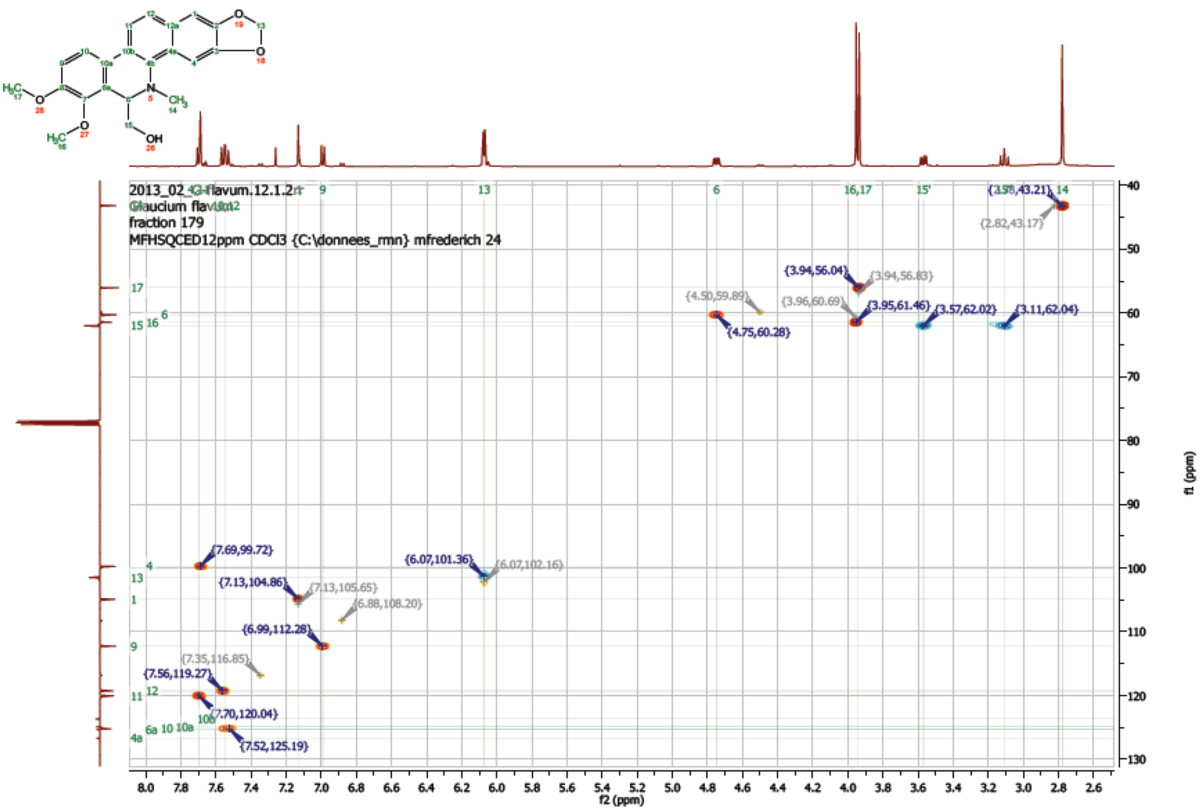

© 2013 by the authors; licensee MDPI, Basel, Switzerland. This article is an open access article distributed under the terms and conditions of the Creative Commons Attribution license (http://creativecommons.org/licenses/by/3.0/).
